# Recent Advances in the Development of 1,4-Cyclohexanedimethanol (CHDM) and Cyclic-Monomer-Based Advanced Amorphous and Semi-Crystalline Polyesters for Smart Film Applications

**DOI:** 10.3390/ma17184568

**Published:** 2024-09-17

**Authors:** Farida Irshad, Nayab Khan, Haidar Howari, Mahvish Fatima, Assad Farooq, Muhammad Awais, Muhammad Ayyoob, Muhammad Qamar Tusief, Razia Virk, Fiaz Hussain

**Affiliations:** 1 Department of Fiber and Textile Technology, University of Agriculture Faisalabad, Faisalabad 36000, Pakistan; faridairshad45@gmail.com (F.I.); knayabrpm@gmail.com (N.K.); assadfarooq@googlemail.com (A.F.); muh.awais@uaf.edu.pk (M.A.); qamartosief@yahoo.com (M.Q.T.); 2 Department of Physics, College of Science, Qassim University, Buraydah 51452, Qassim, Saudi Arabia; hoary@qu.edu.sa; 3 Department of Polymer Engineering, National Textile University, Karachi Campus, Karachi 74900, Pakistan; ch_ayyoob_kamboh@live.com; 4 Department of Biosciences, University of Wah, Rawalpindi 47040, Pakistan; razia.virk@uow.edu.pk

**Keywords:** 1,4-cyclohexanedimethanol, cyclic monomer, polymerization, advanced copolyesters, semi-crystalline polyesters, structure–property relationship, biodegradation, polymeric smart films

## Abstract

Polyester-based advanced thin films have versatile industrial applications, especially in the fields of textiles, packaging, and electronics. Recent advances in polymer science and engineering have resulted in the development of advanced amorphous and semi-crystalline polyesters with exceptional performance compared to those of conventional polymeric films. Among these, 1,4-cyclohexanedimethanol (CHDM) and cyclic-monomer-based polyesters have gained considerable attention for their exceptional characteristics and potential applications in smart films. This review article provides a comprehensive overview of the recent advances in the synthesis, characterization, and applications of CHDM and cyclic-monomer-based advanced polymers for smart film applications. It discusses the structure–property relationships of these innovative polyesters and highlights their unique characteristics, including thermal, mechanical, and barrier characteristics. Furthermore, this article also emphasizes the solution, melt, and solid-state polymerizations of the polymers. Special emphasis is placed on the influence of the addition of a second diol or second diacid on the performance characteristics of synthesized polyesters/copolyesters to explore their versatile industrial applications. Additionally, the impact of the stereochemistry of the monomers is explored to optimize the characterization of polyesters suitable for industrial applications. Furthermore, this article explores the potential of these advanced polyesters to be considered as materials for smart film applications, especially in the field of flexible electronics. Finally, this article examines the challenges and future recommendations for the development of CHDM and cyclic-monomer-based polyesters for smart film applications. It discusses potential avenues for further research, including in-depth studies for the synthesis and characterization of polyesters, the development of sustainable and biodegradable alternatives to cyclic monomers, alternative green approaches for the synthesis of polymers, etc. This review article provides valuable insight for researchers in academia and industry who are working in the fields of polymer science and materials engineering.

## 1. Introduction

Soon after the discovery of high-molecular-weight aliphatic polyesters by Carothers and Hill in 1932, polyesters gained the attention of academia and industrial researchers because of their potential applications [1]. Advanced polymers have gained attention because of their potential applications in the fields of catalysis [2,3], sensors [4], flexible electronics [5], medicine [6], wastewater treatment [7], textiles [8], and packaging [9]. Based on their composition, polymers are broadly classified into two groups: aliphatic and aromatic. Aliphatic polymers contain aliphatic diol and aliphatic diacid parts. However, the low thermal, mechanical, and hydrolytic properties of these materials limit their commercial applications. Aromatic polymers contain aromatic diacid and/or aromatic diol parts, and they are well known for their exceptional thermal, mechanical, hydrolysis, and chemical resistance properties [10,11]. Among a broad range of numerous polymers, poly(ethylene terephthalate) (PET) has found widespread applications in the fields of textiles, electronics, packaging, and molded plastic parts [5,9,12,13,14]. Whinfield and Dickinson reported PET as plastic and fiber in 1949 [15]. Because of the wide range of applications of PET, researchers have a keen interest in synthesizing new copolyesters with superior mechanical and barrier properties compared to those of the parent PET. Because of these commercial applications, the performance demands of PET are increasing rapidly. The suitability of PET as a flexible film substrate for electronic devices [5], textile fibers [8], thermoplastic resins [16], transparent and shrinkable films, and elastomers [17,18] has already been explored. However, we cannot use PET at an elevated temperature because of its high crystallization and low glass transition temperature (T_g_). The barrier properties, especially the moisture barrier property of PET, drop rapidly above its T_g_. So, PET is not suitable for making products that require a moisture barrier at an elevated temperature (above 100 °C). There are two widely used approaches to improve the thermal, mechanical, and barrier properties of PET: first, by introducing some fillers, like graphene [17], silica nanoparticles, or nanotubes [19,20], to the PET resin; second, by controlling the chemical structures of the polyesters themselves. Recently, many efforts have been made to improve the thermal and mechanical properties of copolyesters via the copolymerization and reactive blending of polyesters [21,22,23].

The commercial importance and applications of aromatic polyesters have increased tremendously since the first reported preparation of high-molecular-weight poly(ethylene naphthalene 2,6-dicarboxylate) (PEN) in 1969 [24]. Polyesters with aromatic moieties have attracted attention for decades because of their huge engineering thermoplastic market [25,26,27,28]. PEN is a well-known aromatic polyester with superior barrier and thermal properties (T_g_ = 120 °C for PEN vs. 81 °C for PET). Because of the presence of a double naphthalene ring in the PEN polymer, PEN has superior thermal stability, excellent mechanical properties, very high chemical resistance, and dimensional stability, which make it an ideal candidate as a high-performance material for applications in the engineering thermoplastic market, biosensors, flexible electronic devices, and a wide range of high-temperature applications [29]. The high thermal stability of PEN makes it suitable for high-temperature applications [28,30]. However, the high birefringence of PEN films, the necking phenomenon that occurs during the biaxial stretching of PEN, and the high cost of the monomer, 2,6-naphthalenedicarbxylic acid (NDA), used for the synthesis of PEN hinder the extensive applications of PEN films in versatile areas. Thus, it has gained the attention of scientists and researchers to find alternative ways to utilize the superior barrier, electrical, thermal, and mechanical properties of PEN at a relatively low cost.

A breakthrough in the polyester industry was the discovery of the poly(1,4-cyclohexanedimethylene terephthalate) (PCT) homopolyester prepared from terephthalic acid (TPA) and 1,4-cyclohexanedimethanol (CHDM) in 1959 [31]. Compared to PET, PCT has a higher T_g_ (88 vs. 80 °C) and T_m_ (300 vs. 260 °C), superior chemical resistance, and superior tensile and barrier properties [14]. However, the limited processing window of the PCT homopolymer acts as an obstacle to its commercial applications. The incorporation of varying amounts of CHDM into PET has resulted in the synthesis of a new class of amorphous to highly crystalline copolyesters. These CHDM-based copolyesters rapidly found a strong position in the commercial market of polyesters. Nowadays, CHDM-based copolyesters have a wide range of commercial applications. The performance properties of copolyesters can also be tuned by incorporating the second diacid or second diol.

Numerous pieces of literature are present that emphasize the CHDM diol moiety to enhance the thermal, physical, chemical, and mechanical properties of polymers [14,32,33]. Not only the CHDM content but the stereochemistry of CHDM (cis/trans isomers content) can also improve the resultant properties of the resulting polymers [34,35,36,37]. Trans-CHDM isomers are considered to be more stable than their analogous cis-CHDM isomers [38]. Kibler et al. described that the melting behavior of PCT can be improved by increasing the content of trans-CHDM from 0% to 100% (T_m_ 248 °C vs. 308 °C) [31]. Not only T_m_ but T_g_ of PCT homopolymer is also increased linearly by increasing the trans-CHDM content from 0 to 100% (60 vs. 90 °C). However, the crystallization rate is not similar for different compositions of PCT homopolymer. PCT homopolymer has a limited processing window, which can be controlled by introducing other diacid units into the molecular backbone. When a small amount of isophthalic acid (IPA) is incorporated into the PCT polymer backbone, it widens the processing window at the expense of T_g_ and T_m_ [39]. This modified PCT copolymer is called acid-modified PCT (PCTA).

This review provides detailed information on advanced polyesters based on cycloaliphatic CHDM. The effects of second diacid, second diol, and stereochemistry of monomers are discussed in detail. Mainly, PCT homopolymer, glycol-modified PCT, CHDM-modified PET, acid-modified PCT, and the effect of the stereochemistry of monomers and their potential commercial applications are discussed in detail. A new class of biobased PCT copolymers is also discussed in detail in later sections.

## 2. 1,4-Cyclohexandimethanol (CHDM) and Its Stereoisomers

**Figure 1:**
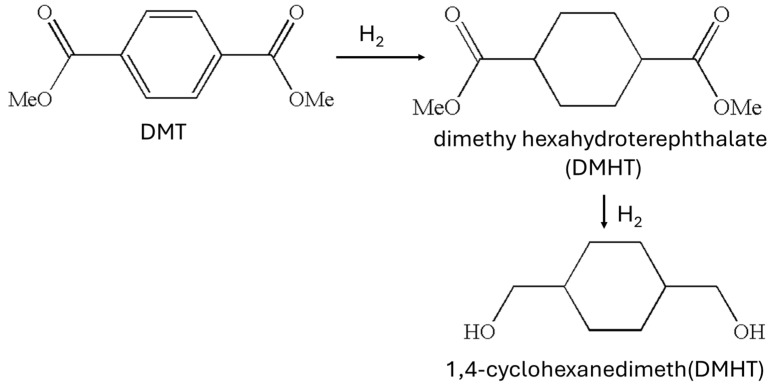
Synthesis of 1,4-CHDM from DMT [40,41,42].

CHDM is a commercially available diol with a cheaper price, and it is widely used for the synthesis of CHDM-based aliphatic and aromatic polyesters. It modifies the unique properties of synthesized polymers. There are three main isomers of CHDM: 1,2-CHDM, 1,3-CHDM, and 1,4-CHDM. The study of 1,2-CHDM and 1,3-CHDM-based polyesters and copolyesters is beyond the scope of this review. Traditionally, CHDM was synthesized on a commercial scale via the hydrogenation of dimethylene terephthalate (DMT) in a two-step process. The scheme for the synthesis of CHDM from DMT is shown in Figure 1 [40,41,42]. In the first step, DMT is converted into dimethyl cyclohexanedicarboxylate (DMCD) by treating hydrogen in the presence of catalyst (Pd) at the temperature of 180 °C, and in the second step, DMCD is reduced into CHDM in the presence of copper chromite catalyst at the temperature of 200 °C.

**Figure 2:**
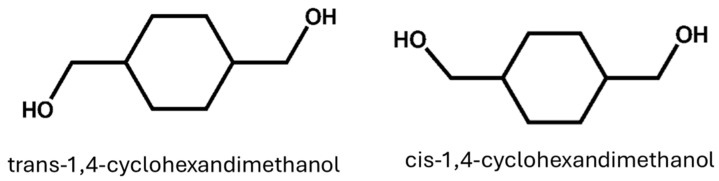
Trans- and cis-isomers of 1,4-cyclohexanedimethanol (CHDM).

In the presence of either a bimetallic nano-catalyst or a supported tri-metallic nanocluster, it has also been proposed to prepare CHDM in a single step by hydrogenating DMT (Ru_5_PtSn). Compared to conventional approaches, these nanocatalysts facilitate the hydrogenation of DMT and allow for a modification reaction with high efficiency in mild conditions (100 °C, 20 bar) [43]. Wei et al. developed a method for preparing DMCD using a continuous hydrogenation process and prepared the CHDM with high activity and selectivity [44]. Recently, PET monomer waste (BHET) has been converted into CHDM in the absence of any kind of solvent utilizing Pd/C and Cu-based metallic catalysts [45]. Yancheng et al. successfully prepared CHDM using bio-based materials. This green synthesis strategy provides a viable alternative to the conventional methods that use hazardous materials [46]. However, some further reports describe the procedure for producing high trans-CHDM [41,47]. Both Eastman Chemical Company USA and TCI Japan are the leading producers of CHDM in the world. CHDM is produced as a cis/trans-isomers 70/30 trans/cis-CHDM isomers combination, and it is used to synthesize all commercial polyesters. The stereochemistry of CHDM has a direct influence on the properties of synthesized polyesters, which will be discussed in detail later. Cis- and trans-CHDM isomers are shown in Figure 2. Furthermore, to support readers’ clarity and ease of understanding, Table 1 highlights the information about the yield rate, catalyst, and summary of the research work, along with relevant references (Table 1).

### 2.1. Aliphatic Polyesters and Copolyesters Containing CHDM and Their Applications

**Figure 3:**
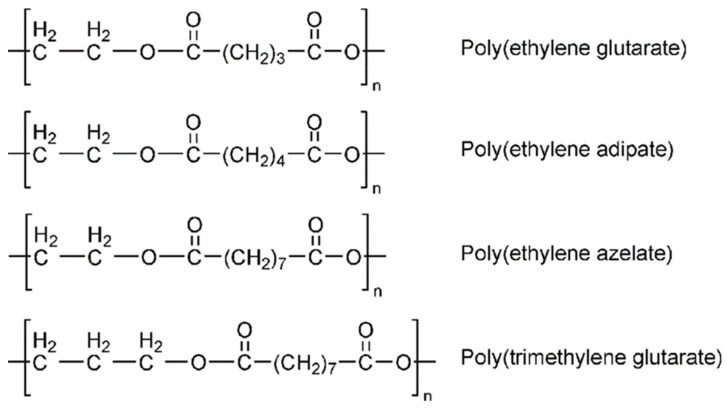
Chemical structure of various aliphatic polyesters [64].

CHDM has been widely employed for the synthesis of biodegradable aliphatic polyesters and copolyesters with numerous uses. Typically, these copolyesters are synthesized using a two-step melt polymerization or enzymatic polymerization process. Two-step polycondensation of HCDM, sebacoyl chloride, and 1,4-cyclohexane diamine resulted in better mechanical, thermal, and biodegradability properties [57]. Tsai et al. recently synthesized a range of biodegradable aliphatic copolyesters using enzymatic polymerization of 1,3/1,4-CHDM with succinic acid and 1,4-butanediol. They were successful in tuning the characteristics of resultant copolymers by varying the amount of CHDM included [58]. Hansen et al. also reported the enzymatic polymerization of aliphatic copolyesters containing CHDM as a diol moiety and succinic acid, atopic acid, and suberic or sebatic acid as a diacid moiety, using Cutinase from Humicola insolens; however, moderate molecular weights of these polymers limits their applications [59]. Barret et al. effectively synthesized a poly(1,4-cyclohexanedimethanol itaconate) thermoset polymer using single-step enzymatic polymerization and analyzed its mechanical and biocompatibility properties. They discovered that this material could be a strong option for future biomaterials [60]. Nowadays, practically all cycloaliphatic polyesters are synthesized from 1,4-CHDM and 1,4-cyclohexanedicarboxyklic acid (CHDA). Many groups have focused on the polycondensation of CHDM with CHDA in order to synthesize high-molecular-weight cycloaliphatic poly(1,4-cyclohexylene 1,4-cyclohexanedicarboxylate) (PCCD). In comparison to the CHDM diol moiety, the cyclohexane ring structure of CHDA remains stable in both cis- and trans-CHDM configurations. As the trans-CHDA isomer content (mole %) increases, the T_g_ and T_m_ of resulting polyesters grow linearly. The trans-CHDA isomers are used to manufacture high-molecular-weight thermoplastic aliphatic polyesters. In contrast to CHDM, cis-/trans-CHDA isomers can easily revert to their equilibrium mixture (68/32%: trans/cis) in the presence of an appropriate catalyst at a high melt polycondensation temperature [39]. Xiaodong et al. synthesized a series of poly(butylene-co1,4-cyclohexanedimethylene carbonate) (PBCC) and investigated the influence of CHDM on the performance properties of synthesized biodegradable PBCC. They observed that the increased CHDM concentration linearly improves the thermal stability and mechanical and heat-distortion properties of PBCC random copolymers [61]. The thermal degradation behavior and other performance attributes of biodegradable aliphatic poly(butylene 1,12-dodecanedioate) random copolyesters were also significantly improved by inserting cycloaliphatic 1,4-cyclohexanedicarboxylic acid units into the molecular backbone. It was discovered that trans-CHDA isomers improve the performance attributes of synthesized aliphatic polymers in a linear manner [62]. Recently, Seul et al. successfully modified the brittle properties of isosorbide (ISB)-based polycarbonate by inserting the second diol. A variety of biodegradable copolycarbonates comprising ISB, cycloaliphatic CHDM, and diphenyl carbonate were synthesized using a two-step melt polymerization technique. A CHDM concentration greater than 50 mole % enhances the ductility of the resulting polyesters. However, when the T_g_ of the synthesized polymer decreases, we increase the content (mole %) of CHDM because the resultant polymer contains less rigid heterocyclic ISB [63]. Figure 3 depicts the chemical structures of various aliphatic polyesters [64].

Brunelle et al. filed a patent for the synthesis of PCCD; they successfully synthesized a cycloaliphatic polymer by optimizing the monomer feed ratio and reaction conditions. Later, they were successful in synthesizing the stereoregular polymer by adjusting reaction parameters such as the temperature, catalyst, and time. The reaction conditions were optimized to prevent the isomerization of trans-CHDA isomers, and they successfully generated PCCD polymers with a molecular weight ranging from 75,000 to 80,000 [65]. High-molecular-weight polyoxaesters with acceptable thermal and hydrolysis properties were synthesized by melt polymerization of CHDM with oligo(ethylene glycol) diacid in the presence of a suitable catalyst. The absorbent polyoxaesters may have biomedical applications as suture coverings, and adhesion-prevention barriers have been proposed [66]. Based on the chemical structure, physical performance, and performance characteristics such as structure integrity, adhesion-prevention barriers, UV resistance, and so on, CHDM-based polyesters have prospective applications in the fields of medical, safety protection, and outdoor applications. Because it is feasible to manipulate the structure of the polyesters by incorporating various suitable diacids or diols, academics and industry researchers are investigating the structure–property relationship of homopolyester and copolyesters. Because of this distinct behavior, polyesters have established a prominent position among other performance polymeric materials [67,68,69].

In short, the literature shows that cycloaliphatic polyesters have been synthesized by employing a variety of monomers and methods. Cycloaliphatic polyesters outperform aromatic polyesters in terms of UV stability, optical performance, and good weatherability properties. These cycloaliphatic polymers could be used as weather-able materials and biomaterials. A detailed study is necessary to delve into the diverse applications of these cycloaliphatic polymers.

### 2.2. Thermally Stable Aromatic Polyesters and Copolyesters Containing CHDM

Thermoplastic polyesters have garnered the attention of academic and industrial researchers because of their vast range of domestic and technical applications [5,9,12,13,14,70]. The synthesis of high-molecular-weight aliphatic polyesters was first reported by Carothers and Hill [1]. However, the inherent poor hydrolytic stability, low glass transition temperature, and melting temperature of aliphatic polyesters have effectively eliminated their commercial applicability. Whinfield and Dickson reported on a new aromatic poly(ethylene terephthalate) (PET) with acceptable T_g_ and T_m_ in 1949 [15]. However, PET’s (81 °C) strong crystallinity and low T_g_ limits its practical applicability at higher temperatures. The mechanical, chemical, and barrier properties of PET can be improved by including rigid cycloaliphatic 1,4-CHDM diol into the backbone of the aliphatic polyester. 1,4-CHDM is accessible commercially in the form of a blend of cis-and trans-CHDM isomers (70/30%). Kibler et al. reported on the synthesis process and thermal characteristics of poly (1,4-cyclohexylene dimethylene terephthalate) (PCT) in 1964. The Eastman Kodak Company successfully synthesized semi-crystalline PCT fiber and marketed it in the fiber industry under the trade name of Kodel for a long period before discontinuing it in 1980 [36]. PCT is now manufactured via two-step melt polymerization with NDA or DMT as a diacid moiety and CHDM as a diol moiety. PCT and its copolyesters have superior thermal, mechanical, chemical, and barrier properties compared to PET [31]. Commercial PCT is highly crystalline with high T_m_ (295 °C), T_g_ (about 90 °C), and thermal degradation stability, and it is less expensive than liquid crystalline polymers (LCP). PCT possesses outstanding thermal, mechanical, and hydrolytic stability characteristics but has a similar flow during molding when compared to traditional PET and PBT polymers. Amorphous copolyesters, including rigid and bulky CHDM, have a wide range of commercial applications, including injection-molded polymers for medical and electronic applications [14,40]. However, both a high crystallinity and high melting temperature (295 °C) of PCT (limited processing window) operate as barriers to melt polymerization. As a result, the commercial applications of PCT copolyesters as films have been limited by their properties. For typical plastic applications, the processability of PCT polymer must be increased by adding it with diacid or diol compounds. The distinctive, unique characteristics and applications of several CHDM-based copolyesters are summarized in Table 2.

## 3. Preparation of CHDM-Based Advanced Polymers

Typically, CHDM-based homopolyesters and copolyesters are made using a polycondensation process. Depending on the types of polyesters, the chemical structure of CHDM and diacid moieties may be changed to optimize the reaction efficiency. Most high-molecular-weight aromatic polymers are synthesized by polymerizing CHDM with diacid at high temperatures and pressures. There have been numerous research studies on the synthesis of CHDM-based polyesters using different techniques [21,59,80,81]; however, the most common techniques are melt polymerization, solution polymerization, and ring-opening polymerization. The following section provides deeper details about various synthesis strategies

### 3.1. Solution Polymerization

**Figure 4:**
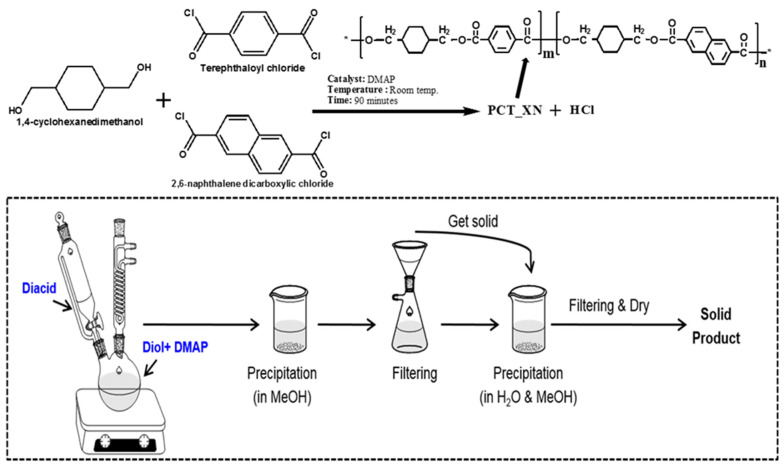
Schematic diagram for the solution polymerization of PCTN copolyester. * Indicates the open ends of a single monomer of PCTN copolyester ready to react with other monomers to build a long chain polymeric chain.

Almost all polymers are now synthesized using a complicated two-step melt polymerization process. The first process involves the synthesis of pre-polymer by esterification or a transesterification reaction, and in the second step, known as polycondensation, the synthesized prepolymer reacts with diol at relatively high temperatures and pressures. A product removal setup is also included at both stages of polymerization [21,82]. Due to the severe conditions used during melt polymerization, a stoichiometric imbalance is observed due to the degradation and sublimation of monomers, which causes an increase in side reactions and a decrease in reaction efficacy [83,84]. Some of the issues related to melt polymerization are resolved by utilizing appropriate metallic catalysts [85,86]. However, titanium-based catalysts, which are thought to be the most effective of all metallic catalysts, produce a yellow tint in the synthesized product, whilst antimony-based catalysts are involved with some toxicity problems [87,88]. Generally, an extra thermal stabilizer with metallic catalysts is necessary to prevent polymer degradation during polycondensation and the following process, which results in increased cost [89]. It is worth noting that no polymer degradation or product discoloration occurs during the solution polymerization reaction [90,91]. The primary challenge with solution polymerization is the selection of a pure solvent that promotes monomer solubility and the facile recovery of synthesized polymer products. A reproducible one-step solution polycondensation process is widely known for producing pure and well-defined polymers. A schematic diagram of solution polymerization used for the synthesis of cyclic monomers—CHDM, 2,6-naphthalene dicarboxylic and terephtalloyl chloride-based advanced Poly(1-4,cyclohexane dimethylene terephthalate-co-naphthalene dicarboxylate) PCTN copolyester is shown in Figure 4 [92].

### 3.2. Melt Polymerization

CHDM-based aliphatic and aromatic polyesters and copolyesters are preferably synthesized using the two-step melt polymerization technique. Esterification/transesterification is typically the initial step in polymer synthesis, and it is carried out at relatively low temperatures and pressures depending on the monomers utilized. Typically, an excess amount of diol moiety to diacid moiety (1:1.2–2.2) is employed during the synthesis process, resulting in short oligomers during the esterification step. Short oligomers react under high pressure and temperature to form high-molecular-weight polymers, a process known as polycondensation. The byproducts from each process are removed individually.

**Figure 5:**
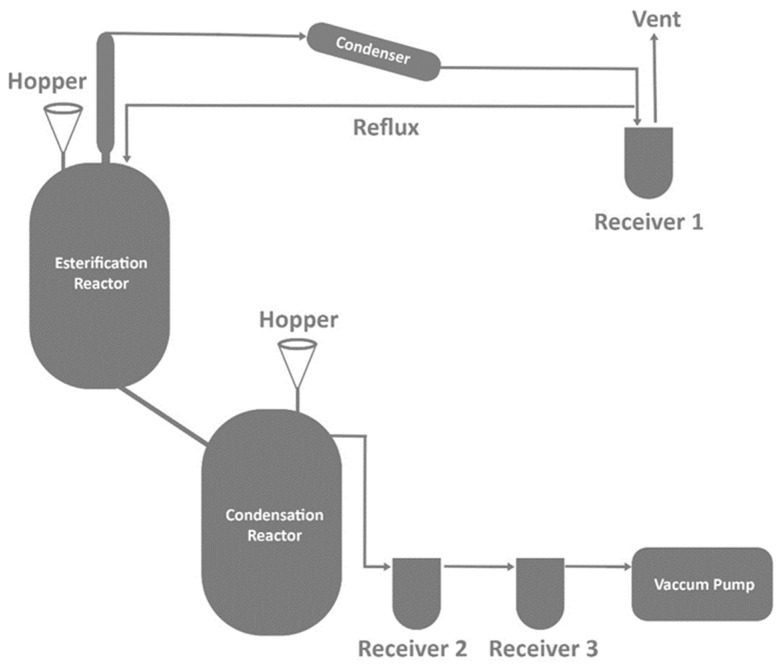
Pilot-scale melt polymerization reactor [33].

Two-step melt polymerization techniques have been used to synthesize a wide range of aromatic and aliphatic polyesters and copolyesters [23,32,35,37,49,83]. The optimal reaction conditions, such as temperature, time, and pressure, are chosen based on the polymers to be manufactured. Catalysts and thermal stabilizers are carefully selected because they have a direct influence on the color of the finished product. Figure 5 shows the typical melt polymerization reactor that is used for the melt polymerization of polyesters.

### 3.3. Ring-Opening Polymerization

Nowadays, cyclic oligoesters’ ring-opening polymerization (ROP) is gaining attention. ROP offers some advantages over conventional melt polymerization. ROP, unlike melt polymerization, occurs at low temperatures and pressures. Furthermore, no by-products, such as water or methanol, are produced during the synthesis of polyesters using ROP [93,94]. A lot of literature exists that focuses on the synthesis of polyesters and copolyesters using ROP [88,93,94]. Aromatic polyesters and copolyesters have been synthesized via ROP [80,95,96]. CHDM-based poly(1,4-cyclhexylenedimethylene terephthalate) cyclic oligomers were synthesized in solution from CHDM and TPC and separated as a mixture of oligomers of various sizes using high-performance chromatography. The ROP of these oligomers was carried out at 310 °C for 30 min in the presence of antimony oxide for the synthesis of a high-molecular-weight PCT homopolymer [96].

Nathalie et al. used ROP to synthesize a series of poly(ethylene-co-1,4-cyclohexanesimethylene terephthalate) copolyesters (coPE_x_C_y_T), altering the ET/CT monomer ratios ranging from 90/10 to 10/90. Oligomers for the synthesis of coPE_x_C_y_T copolyesters were synthesized by the copolycondensation of PCT and PET homopolymers. The results indicated that synthesized coPE_x_C_y_T copolyesters were random copolymers with high molecular weights [80].

### 3.4. Solid State Polycondensation (SSP) of Polyesters and Copolyesters

The need for high-molecular-weight and low-cost polyesters and copolyesters for engineering plastic applications is growing, indicating the necessity to synthesize tiny particles. Solid state polycondensation (SSP) can be used to produce polyesters with thermally unstable components that can disintegrate under harsh melt polymerization conditions. Isothermal treatment at temperatures ranging between a cold crystallization temperature (T_c_) and melting temperature (T_m_) in the solid state increases the molecular weight of melt polymerized polyesters and copolyesters. This process is known as solid state polycondensation. The molecular weight of polyesters and copolyesters influences their performance properties, such as thermal stability, tensile strength, fatigue behavior, and hydrolytic stability [97]. SSP allows for the production of high molecular weights of polyesters and copolyesters that would otherwise be impossible to achieve using melt polymerization processes. Melt polymerization produces an intrinsic viscosity (IV) of 0.58–0.68, whereas, for technical applications (such as film, bottle, tire cord, seat belt, airbags, etc.), an IV of typically between 0.70 and 1.20 is required. The SSP process is popular because it eliminates the issues associated with melt polymerization. Some issues arise during the polycondensation of viscous polymers in the melt phase. During melt polycondensation, higher-IV polyesters with increased viscosity are difficult to stir, and thermal degradation occurs at higher temperatures, resulting in low-molecule polymers. It is extremely difficult to extract volatile by-products from highly viscous polymers. This procedure is also carried out at high temperatures, and the vacuum increases the expense of the product. Furthermore, the thermal degradation of the polymer occurs due to the result of undesired side reactions, which hinder the growth of molecular chains. As a result, the melt viscosity and molecular weight of the synthesized product decrease. SSP addresses all of the limitations of melt polycondensation by operating under comparatively mild conditions [98]. SSP is of relevance because of the increasing need for polyesters and copolyesters in widespread areas requiring high molecular weights. Furthermore, certain monomers and polymers demand mild conditions that cannot be met in the melt phase. As a result, the SSP technique is preferred for the production of high-quality homopolyesters and copolyesters with improved performance properties.

On an industrial scale, continuous or discontinuous SSP processes are carried out either in a vacuum or supported with an inert gas flow. Another form of SSP, known as the suspension process in a swollen state, can produce higher-molecular-weight polyesters [99]. Generally, large-scale manufacturing of polyesters or copolyesters suited for high-tech applications is performed in a continuous process. Discontinuous SSP is carried out in a tumble dryer, and it is thought to be versatile and simple. It enables the successful manufacturing of specialty materials on a small scale, particularly for engineering plastics [100]. However, the reactor’s small volume (44 m^3^) restricts its use. Different SSP parameters, including temperature, residence time, gas type, gas purity, and gas speed, are paid a lot of attention since they have a direct impact on the final quality of SSP [98,101,102]. The prepolymer’s end group concentration, catalyst, molecular weight, homogeneity, and pallet size all have an impact on the final product’s quality [103]. The chemical structures of monomers in polymer synthesis affect the reaction rate, which in turn influences the molecular weight of the synthesized polymer [104]. The SSP reaction follows the classical thermodynamics, second-order chemical kinetics, and diffusion rates. During the process, the tiny chains join together, and then reactive end groups diffuse into the amorphous regions of semi-crystalline polyesters. As a result of SSP, amorphous regions of the result high-quality product are decreased, and the regular arrangement of molecular chains is increased [105,106]. The consequent high product quality and performance properties make SSP an attractive approach for producing high-molecular-weight polymers suited for use as engineering plastics in versatile areas. A summary of the various polymer synthesis techniques, along with pros and cons, are given in Table 3.

## 4. Synthesis of Cyclic Compound-Based Advanced Hompolyesters and Copolyesters

### 4.1. Synthesis and Properties of 1,4-Cyclohexanedimethanol (CHDM)-Based Conventional Homopolyesters (PCT and PCN)

**Figure 6:**
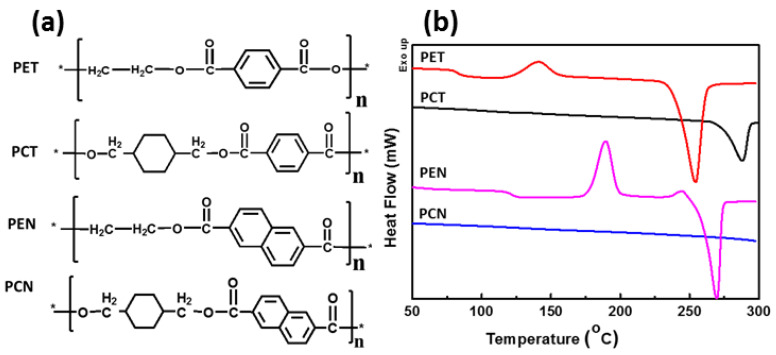
(**a**) Chemical structure of PET, PCT, PEN, and PCN homopolyesters; (**b**) DSC thermograms of PET, PCT, PEN, and PCN homopolyesters. * Indicates the open ends of a single monomer of each homopolyester, ready to react with other monomers to build a long chain polymeric chain.

The synthesis and application of CHDM-based PCT homopolymers was first discovered by Kibler et al. in 1949 [36]. This polyester was first synthesized by a two-step melt polymerization, and its applicability as a fiber was investigated. On a commercial scale, PCT is synthesized by melt polymerization of NDA or DMT with CHDM in the presence of an appropriate catalyst and stabilizer. Because of the high melting point of PCT homopolymer, polycondensation occurs at a range of temperatures higher than 300 °C [36]. SSP can further boost the molecular weight of the synthesized PCT polyesters [21]. Compared to PET, PCT has greater T_g_ (88 vs. 80 °C), T_m_ (300 vs. 260 °C), superior chemical resistance, attractive tensile properties, and barrier properties [14]. Due to its superior thermal characteristics, high clarity, and improved molding characteristics, PCT is used as an injection-molded polyester for developing electronic and automotive parts [39]. However, the limited processing window of the PCT homopolymer makes it unsuitable for a wide range of commercial applications. The addition of various amounts of second diol or second diacid into the PCT homopolymer effectively broadens the processing window, resulting in the synthesis of a new class of amorphous to highly crystalline copolyesters with a strong position as performance materials in the commercial market of polyesters. The replacement of acyclic aliphatic diols with stiff cycloaliphatic CHDM diol leads to the synthesis of polyesters with better thermal properties [39,40]. The chemical structures of PET, PCT, PEN, and PCN are shown in Figure 6a. DSC thermograms of these homopolyesters clearly reveal that stiff cyclohexene units (CHDM) improve the T_g_ and T_m_ of the resulting polyesters Figure 6b.

The type of diol and diacid moieties have a direct impact on polyester performance qualities. A list of comparative properties of PET, PCT, PEN, and PCN is given in Table 4. However, the film properties of PCN polyester are not addressed because the synthesis of PCN resin with such a high melting temperature and limited processing window is unsuitable for industrial usage. The results show that CHDM units enhance the thermal properties of polyesters when compared to those containing EG units. Similarly, rigid and thermally stable naphthalene units improve the mechanical and thermal properties of polyesters when compared to their analogous polyesters containing terephthalate units [24,26,36,39,40].

### 4.2. Second Diol Modified PCT Copolyesters

**Figure 7:**
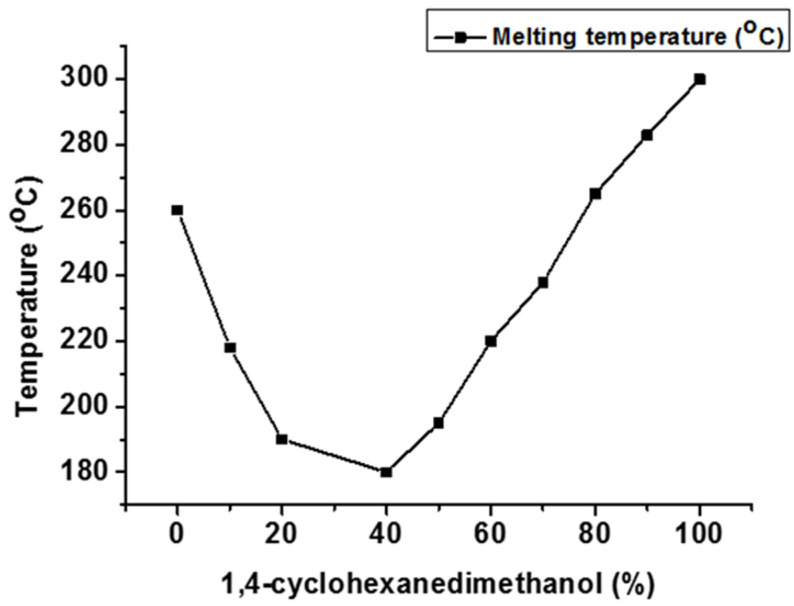
Effect of 1,4-CHDM on T_m_ of CHDM-modified PET copolyester [39].

Modifying the diol moiety allows for the synthesis of novel polyester-based materials with correct molecular design. Cycloaliphatic 1,4-cyclohexanedimethanol (CHDM) is easily incorporated into the polyesters during melt polymerization. The non-planar ring structure of CHDM enhances the thermal stability and mechanical and barrier properties of the resultant poly(cyclohexane 1,4-dimethylene terephthalate) (PCT) [39]. However, there are significant processing problems associated with the synthesis of PCT that can be addressed by adding a second diol into the polymer backbone itself (Figure 7). T_m_ of CHDM-modified PET polyester drops initially until it hits the eutectic point around 40 mol% 1,4-CHDM, after which it begins to grow exponentially as the CHDM level increases. At the eutectic point, PET and PCT crystals coexist in the copolyester. The findings revealed that the rigid and cyclic aliphatic CHDM increase the rigidity and regularity of polymer backbones [39].

**Figure 8:**
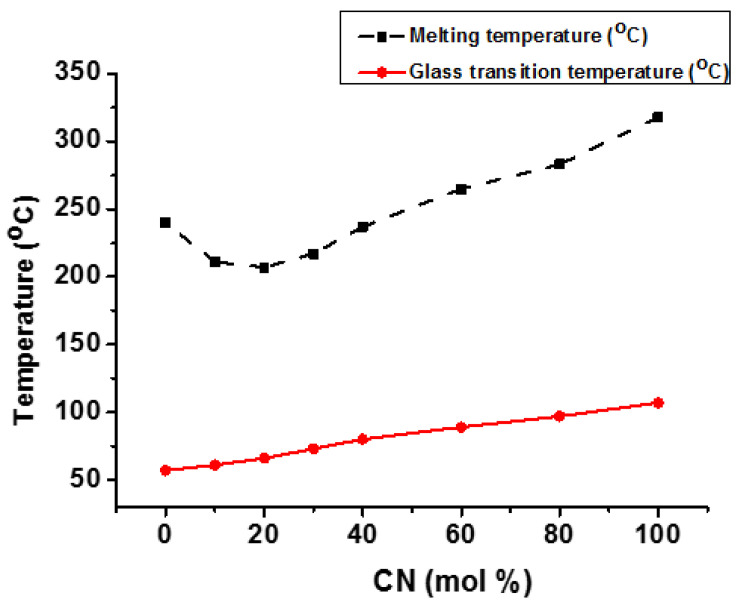
Effect of composition on T_g_ and T_m_ of PBN-co-CN copolyester [112].

Jo et al. synthesized and investigated the crystallization behavior of poly(m-methylene 2,6-naphthalate-co-1,4-cyclohexanedimthylene 2,6-naphthalate) (m = No. of methylene groups). They discovered that poly(ethylene 2,6-naphthalte-co-1,4-cyclohexanedimethanol 2,6-naphthalate) (PEN-co-CN) had an amorphous structure in the center of copolymer composition, while poly(butylene 2,6-naphthalate-co-1,4-cyclohexanedimthylene 2,6-naphthalate) (PBN-co-CN) and poly(hexamethylene 2,6-naphthalate-co-1,4-cyclohexanedimthylene 2,6-naphthalate) (PHN-co-CN) had very clear melting points and also showed sharp diffraction peaks across the entire range of copolymer composition. Additionally, (PBN-co-CN) exhibited eutectic melting behavior, with T_g_ increasing linearly as CN % rose (Figure 8) [112]. However, in the case of (PHN-co-CN) copolyester, both T_g_ and T_m_ grew linearly as CN units increased, indicating that this copolymer had an isomorphic crystallization nature. These results show that BN and HN units can co-crystallize together while EN and CN cannot. This trend could be explained by the fact that BN and HN units have equivalent densities, volumes, and repeating unit lengths, whereas EN and CN units do not [112].

**Figure 9:**
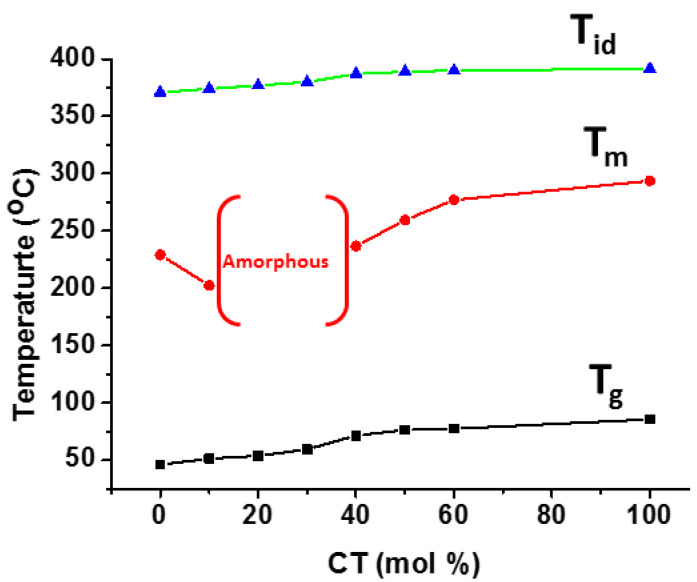
Effect of composition on T_g_ and T_m_ of PTCT copolyester.

Aromatic poly(trimethylene-co-1,4-cyclohexanedimethylene terephthalate) (PTCT) with a random microstructure was synthesized by the two-step melt polymerization of 1,3-propanol, CHDM, and DMT [113]. At 42 mole % of CHDM units, both tri-methylene terephthalate (TT) and cyclohexylene dimethylene terephthalate (CT) coexist in the PTCT copolyesters. At the same time, copolyesters with less than 35 mole % of CT content crystallize in a PTT-type lattice, whereas those with more than 42% CT content crystallize with a PCT-type lattice. The thermal degradation behavior and other thermal parameters of the synthesized copolyesters were improved by increasing the CT content (mol %), as shown in Figure 9.

A variety of copolyesters derived from PCT and PET homopolymers were identified. Copolyesters were synthesized by polymerizing TPA with different amounts of CHDM and EG, resulting in copolyesters having 12, 31, 32, 61, 70, and 81 mol % CHDM. It was discovered that the free volume and gas permeability of synthesized copolyesters increased linearly with the CHDM concentration [114].

If the EG is replaced with the CHDM, poly(ethylene glycol-co-1,4-cyclihexanedimethanil terephthalate) (PETG) is formed. The inclusion of CT units into the backbone of PET increases the alkali resistance of PETG copolyesters. The amorphous regions of PET and PETG polyesters were more susceptible to attack by the alkali than crystalline regions. Furthermore, hydroxyl anions caused corrosion to crystals without altering the crystalline structure of synthesized polyesters [115].

When the CHDM of PCT is substituted with hexanediol, the resulting poly(1,4-cyclohexylenedimethylene terephthalate-co-hexamethylene terephthalate) [P(CT-co-HT)] are random copolyesters with iso-dimorphic co-crystallization behavior. DSC and WXRD results confirmed that synthesized copolyesters are crystalline in nature with an eutectic point at 80 mol% of HT when a crystal transition from a PCT-type crystal to PHT-type crystal occurs [116].

### 4.3. Second Diacid-Modified PCT Copolyesters and Their Applications

**Figure 10:**
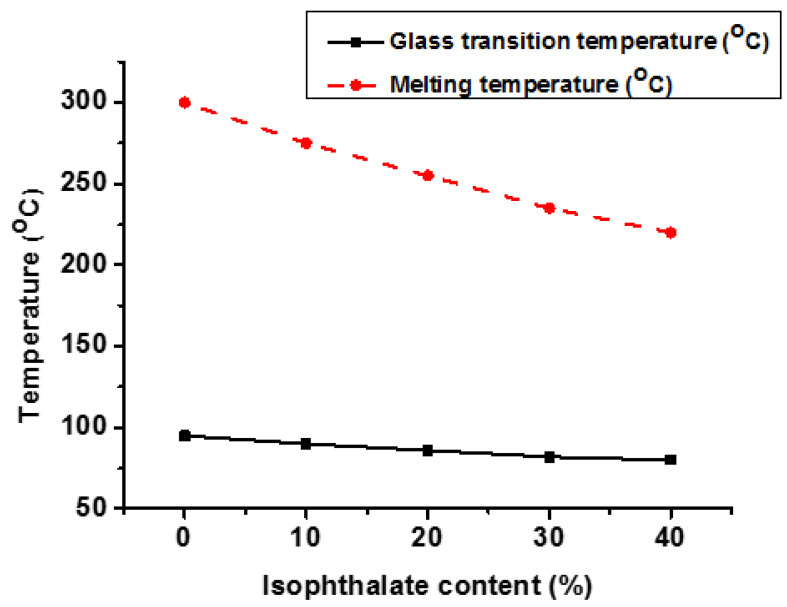
Effect of isophthalate content (%) on T_g_ and T_m_ of PCT copolyester [39].

As previously stated, CHDM and TPA-based PCT homopolymers have a limited processing window, which can be regulated by increasing other diacid units in the molecular backbone. When a small quantity of isophthalic acid (IPA) is added to the PCT polymer backbone, it expands the processing window at the expense of T_g_ and T_m_ [39]. This PCT copolymer is known as acid-modified PCT (PCTA). Figure 10 demonstrates that as the IPA content percentage increases in PCT, both T_g_ and T_m_ are decreased. However, T_m_ decreased more than T_g_. It was also found that at higher concentrations (more than 40%) of IPA, completely amorphous copolymers were obtained [39,117]. These transparent amorphous copolymers have excellent mechanics, hydrolysis, and chemical resistance. PCTA has several attractive performance features, which can be due to the presence of tough and hydrophobic CHDM units. These copolymers can be melt-processed without pre-drying and hold a very strong position in the plastic industry due to their performance properties. PCT_XA_X30 (30% IPA) copolyesters have good physical, thermal, and mechanical properties equivalent to glycol-modified PCT and CHDM-modified PCT [39].

The thermally stable naphthalene unit (NDA) was identified as a modifier for acid-modified copolyesters (PCTAs). The addition of a modest amount of diacid into the polymer backbone effectively widens the processing window of PCT polyester by lowering its T_m_. These semi-crystalline copolyesters (PCTN) are durable and transparent resins. The impact of increasing levels of naphthalene content (mol %) on the thermal characteristics (T_g_, T_m_, ∆H_m_) and degradation behavior of PCT is summarized in Table 5 [21,92]. As indicated, the thermal degradation stability and T_g_ of the synthesized PCTN copolyesters rose linearly as the number of naphthalene units increased. However, the T_m_ of copolymers first decreases until it reaches the eutectic point (36 mole % of naphthalene), after which it begins to increase by increasing the naphthalene concentration. At the eutectic point, PCT and PCN crystals coexist in the copolymers, and after this point, the main crystal structure is dominated by the PCN-type crystal that enhances the thermal, mechanical, and physical properties of copolymers [21,82,117].

**Figure 11:**
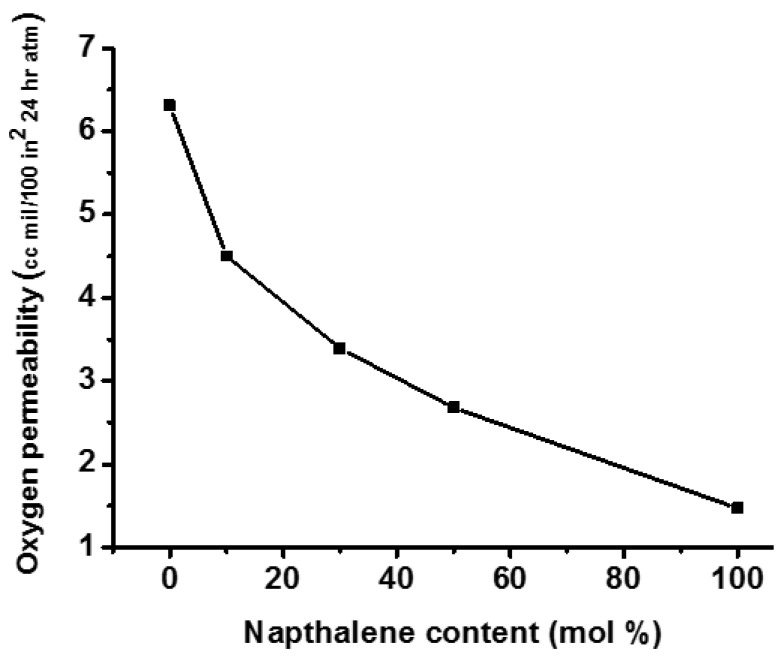
Effect of naphthalene content on the gas barrier properties of PCTN [120].

Many patents have been filed for the synthesis of CHDM-based thermally stable copolyesters containing naphthalene units. These copolyesters demonstrated unusually high T_g_, T_m_, and thermal stability [36,118,119]. The incorporation of naphthalene units into PCT results in the synthesis of a new class of PCTN copolyesters with remarkable gas barrier properties that are suitable for packaging applications. Sublett demonstrated that the gas barrier properties of PCTN copolyesters synthesized using CHDM and containing about 92/8 cis/trans isomers may be successfully regulated by controlling the amount of incorporated naphthalene units (Figure 11) [120]. In general, PCTN copolyesters outperform ordinary polymers in terms of barrier and thermal properties. It is evident from Figure 11 that the addition of cyclic rings of naphthalene in the backbone of PCT homopolyester resulted in the significant improvement in the barriers characteristics of the resultant PCTN copolyester. It improved behaviour is attributed to more stable structure of the PCTN copolyesters that is caused by the naphthalene rings.

**Figure 12:**
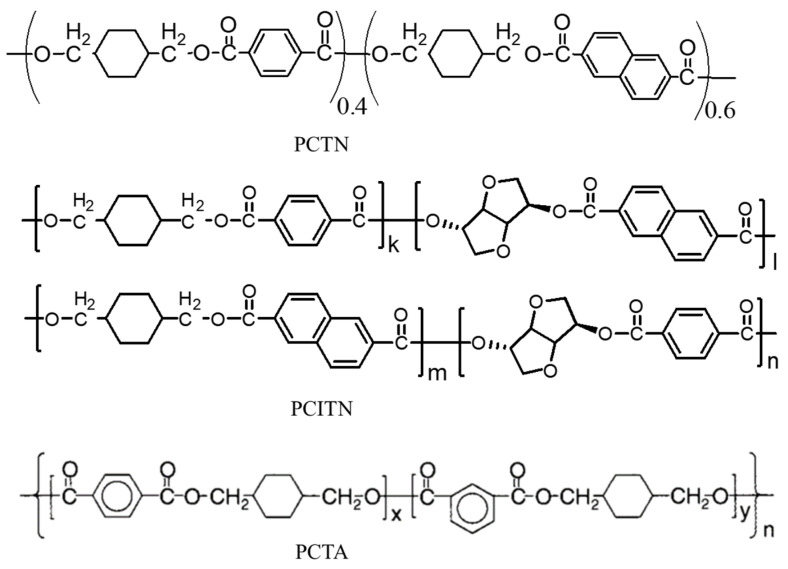
Chemical structure of acid-modified PCT copolyesters (PCTN, PCITN, and PCTA).

Researchers have turned their focus to bio-based polymers in response to serious environmental pollution and the rapid depletion of oil resources. Extensive research has been conducted to develop monomers from renewable resources that could potentially replace the monomers derived from petrochemical resources [121,122,123]. The NDA as a second diacid moiety and biobased isosorbide as a second diol moiety can also be included in the backbone structure of PCT homopolyester, resulting in a quadri-polymer with superior thermal, barrier, and degrading properties [78,124]. The chemical structure of several acid-modified PCT copolyesters is shown in Figure 12.

Compared to NDA units, 1,4-cyclohexanedicarboxylic acid (CHDA) (95% trans isomers), as a modifier polyester, retained the toughness of the parent polyester while lowering the T_g_. The addition of a modest amount of CHDA units significantly reduces the T_g_ and T_m_ of PCT homopolyester. However, high-molecular thermoplastic copolyesters are synthesized by integrating high trans-CHDA isomers, as they improve the thermal characteristics of polyesters [125].

Copolyesters derived from the mixture of EG and CHDM, as well as NDA and SA, have been reported. The heat-distortion temperature, T_m_, and degradation behavior of the synthesized copolyesters were discovered to be dependent on the amount of second diol (CHDM) and second diacid (NDA, SA) introduced into the copolyesters. It is crucial to note that the incorporation of 30 mol % or higher amount of CHDM resulted in the synthesis of amorphous copolyesters. The thermal properties (T_g_, T_m_, and IV) of acid-modified copolyesters are presented in Table 6 [36,82,126].

### 4.4. Effect of Stereochemistry of Monomers on Synthesized Polyesters

**Figure 13:**
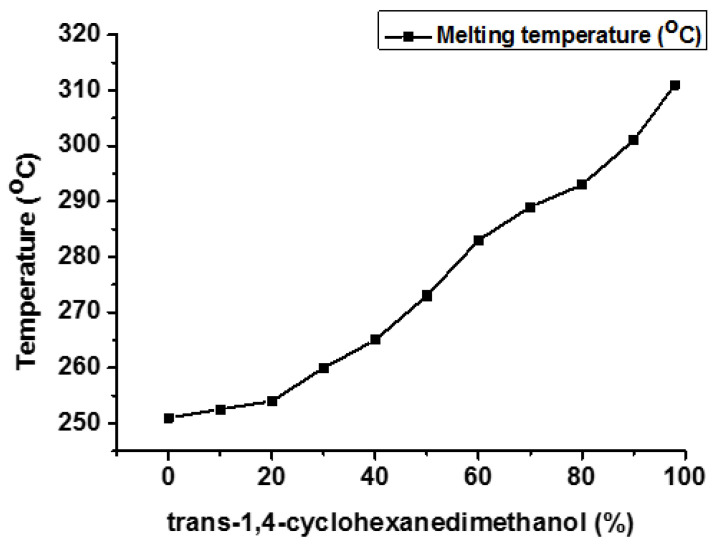
Effect of trans-1,4-CHDM on T_m_ of PCT homopolyester [31].

There is much literature available that emphasizes the use of CHDM diol moieties to improve the thermal, physical, chemical, and mechanical properties of polymers [14,32,33]. Not only does the CHDM content but also the stereochemistry of CHDM (cis/trans isomers content) improve the overall characteristics of the resulting polymers [34,35,36,37]. Kibler et al. reported that the melting behavior of PCT can be improved by increasing the concentration of trans-CHDM from 0% to 100% (T_m_ 248 °C vs. 308 °C) [31] (Figure 13). Not only T_m_ and T_g_ of PCT homopolymer grow linearly when the trans-CHDM content increases from 0 to 100% (60 vs. 90 °C). However, the crystallization rate is not comparable for different compositions of PCT homopolymer.

**Figure 14:**
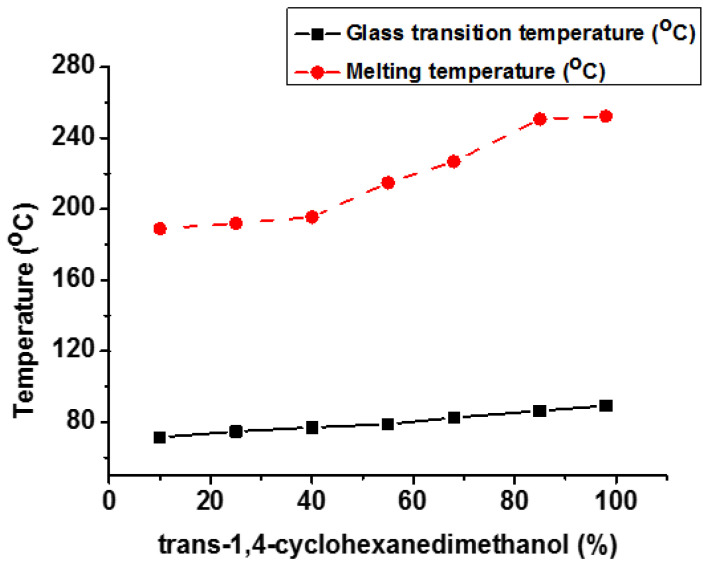
Effect of trans-CHDM content (%) on T_g_ and T_m_ of PECF copolyesters [127].

Wang et al. studied the relationship between the stereochemistry of 1,4-CHDM and the performance attributes of bio-based poly(ethylene 2,5-furandicarboxylate) (PECFs) [127]. It was discovered that trans-CHDM isomers significantly improve the T_g_, T_m_, and T_c_. Figure 14 depicts the influence of trans-CHDM on T_g_ and T_m_ of PECF copolymers. The polymer crystal structure was changed from amorphous to highly crystalline by increasing the percentage of trans-CHDM isomers from 25% to 98%. The mechanical (tensile strength, tensile modulus) and gas barrier (oxygen and carbon dioxide) properties were dramatically improved by adding trans-CHDM isomers in the synthesized bio-based polymer. Stable and stretched trans-CHDM isomers increase the symmetry of polymeric chains and help to create stable crystals.

In a study by Berti et al., it was discovered that by adjusting the cis/trans ratio of the diacid moiety in copolyester, it is possible to control both the thermal properties and crystal structure of the material [128]. T_g_, melting behavior, and crystallization behavior of poly(1,4-cyclohexanedimethylene 2,6-naphthalene) (PCN) can be used to tune by controlling the cis/trans configuration of CHDM [129]. Superior thermal, mechanical, and barrier characteristics of copolyesters containing high trans-CHDM content can be attributed to the more symmetrical structure of CHDM, which facilitates the formation of stable crystal structures. Meanwhile, cis-CHDM obstructs the development of stable structures [35,37,125,129,130]. The comparative properties of conventional PET, PCT, PEN, and PCN homopolymers are listed in Table 7 [71,131,132,133,134].

## 5. Polymeric Substrates for Flexible Electronics

PET, PEN, and PCT homopolymers, as well as related copolyesters, are not novel polymers, but they have drawn significant attention from researchers due to their remarkable thermal, chemical, mechanical, gas barrier, and hydrolysis characteristics. There has been a huge effort to introduce the synthesis of new polyester with comprehensive properties of three polyesters stated so far, namely, PET, PCT, and PEN. In this study, a variety of poly(1,4-cyclohexylenedimethylene terephthalate-co-1,4-cyclohexylenedimethylene 2,6-naphthalenedicarboxylate) (PCTN) copolymers having CHDM as a diol and TPA and NDA as diacids were attempted. The importance of the CHDM configuration (cis/trans isomer) on the hydrolytic stability and thermal, mechanical, and barrier properties of copolyesters was investigated in depth. Based on this work, we will be able to develop a polymer with unique applications as a performance material in the field of textiles, the packaging industry, printing and embossing films, and electronics.

Researchers are currently focusing heavily on flexible electronics. These electronics are thin, lightweight, durable, conformable, and rollable. Furthermore, organic light-emitting diode (OLED) materials and the active matrix of thin film transistor (TFT) arrays can be laid down using solution casting and inkjet printing of plastic-based substrate. As a result, adopting roll-to-roll processing in large volumes effectively reduces processing costs. To replace glass, flexible plastic substrate materials exhibit performance properties comparable to glass, such as a smooth surface, chemical resistance, barrier, thermal stability, and very low coefficient of linear thermal expansion (CLTE). However, flexible glass is fragile by nature, and its handling is also very difficult. Until now, no plastic substrate materials have been reported to meet the performance properties required for flexible substrate materials for organic light-emitting displays (OLEDs). The surface roughness, clarity, thermal, thermomechanical, chemical, mechanical, electrical, and magnetic properties are major properties required for flexible substrate materials suitable for displays. In addition to bottom-emitting displays, the substrate materials for OLEDs must have excellent optical properties. The thermal properties of polymeric substrates (CLTE, T_g_, and T_m_), particularly T_g_, must be compatible with the device production process temperature (T_max_). The thermal mismatch between flexible polymeric substrates and device films may cause device breakage. One of the primary concerns with the flexible substrate materials for OLEDs is their dimensional stability. It should not contaminate the device and possess strong barrier properties. It should be inert against the chemicals employed in device manufacture [135,136]. The standard moisture vapor transmission rate and oxygen permeability of flexible substrate materials for displays are 10^−6^ g/m^2^/day and 10^−5^ cm^3^/m^2^/day, respectively [138]. The good mechanical qualities of the substrate support the device and strengthen its impact resistance. Eclectically insulating polymeric substrates improve the device efficiency by minimizing coupling capacitances.

Previously, semi-crystalline thermoplastic homopolymers such as PEN and PET; non-crystalline thermoplastic polymers polycarbonate (PC) and polyethersulphone (PES); and high-T_g_ materials such as polyarylate (PAR), polyimide (PI) and poly cyclic olefin (PCO) were thought to be good candidates for flexible substrates. PC, PES, PAR, and PCO polymers are more transparent and have a higher T_g_ than PET and PEN. Compared to PET and PEN, these copolyesters have limited chemical resistance and a high coefficient of thermal expansion (CTE). PET, PEN, and PI have desirable performance characteristics. They have reasonably low CTE (15, 13, and 16 ppm/°C, respectively), high mechanical properties, and sufficient chemical resistance for the process. PET and PEN have good optical (transmittance > 85%) and water-absorption (0.014%) properties. However, the low thermal properties of PET and PEN have limited their practical applications in the field of flexible electronics. In contrast to PEN, PI has excellent thermal characteristics, but its yellow color and water absorption properties limit its applicability. At the same time, the water and oxygen permeability rates of conventional materials used as a substrate for flexible displays are 1–10 g/m^2^/day and 1–10 cm^3^/m^2^/day, respectively [136]. So far, no polymer has been reported to meet the rigorous requirements (water and oxygen permeability for organic light-emitting diode (OLED) displays. A comparison of different properties of conventional materials (PET, PEN, glass, steel, and PI) used as base substrates for flexible electronics is summarized in Table 7 [135,136,137].

In recent years, there has been a significant amount of advanced research into the synthesis and development of sophisticated polymer substrates as a smart film for flexible electronics. Currently, PI is the most extensively used polymeric substrate for flexible electronics. The performance parameters, including physical, thermal, mechanical, and barrier characteristics of the randomly oriented, uniaxially oriented, and biaxially oriented advanced polymeric substrates in contrast to conventional PI polymeric substrates, are summarized in Table 8 [33,78,79,124,139,140]. It is crucial to note that there is a potential to build transparent polymeric substrates with good barrier, optical, and thermal characteristics that can replace the yellow PI, which has higher water absorption. Such advanced polymer substrates can be used not only as substrates but also can be used at the top of flexible electronics. Such flexible polymeric smart films with low birefringence and high transmittance also have the potential to replace the brittle glass in flexible displays.

## 6. Future Recommendations for 1,4-Cycloheanedimethanol (CHDM) and Cyclic Monomer-Based Advanced Polyesters

Advanced polymeric materials, including CHDM and cyclic monomers such as TPA, NDA, IPA, ISB, etc., have established a strong position among polymers, laying the groundwork for the creation and characterization of innovative materials with diverse industrial applications. However, in order to further research in this sector, numerous crucial aspects must be investigated. To begin, a thorough study into the synthesis and characterization of novel cyclic monomers with superior characteristics should be conducted in order to expand the library of accessible monomers that serve as the basic building blocks of resulting polymers. It is possible to achieve this by finding alternative synthesis methods, catalysts, and optimized reaction conditions to support the reaction efficiency and yield of the reaction. Additionally, a thorough investigation of the structure–property relationships of the advanced polyesters is required to understand the impact of various monomer structures on the resulting polymers. This would help to design and optimize polyesters with regulated performance characteristics, including mechanical, thermal, and barrier properties for specified applications.

Not only is basic monomer research vital, but research on sustainable and environmentally friendly approaches for the synthesis and processing of CHDM-based polyesters is also very important. The use of biobased raw materials and green synthesis methodologies such as biobased or bio-inspired approaches should be explored. This may include using biobased renewable materials, producing efficient and selective catalysts, and implementing energy-efficient reaction conditions. The development of a recycling procedure, as well as extensive investigation into the biodegradation behavior of these materials, would help to ensure their sustainability and circularity. Collaboration between industry and academia is essential for the commercialization of these materials. Industrial collaborators can provide useful information about the scalability and commercial viability of the produced polymeric materials. The collaborators can also assist in identifying unique industrial requirements and challenges for the development of specialized materials that can establish a strong position in the commercial sector. Furthermore, interdisciplinary collaborations, including researchers and scientists from polymer chemistry, material science, engineering, and industrial design fields, are required. This can lead to holistic approaches in material development, covering not only the synthesis but also the processing, functionalization, and application aspects. Finally, advanced applications of polyesters in a variety of disciplines, including automotive, electronics, textile, packaging, and biomedical sectors, should be pursued in order to fully realize the potential of these advanced polyesters in diverse industries.

## 7. Conclusions

In conclusion, recent breakthroughs in the development of 1,4-cycloheanedimethanol (CHDM) and cyclic monomer-based advanced polyesters have revealed that these advanced copolyesters have the potential to be employed for smart film production with a wide range of industrial applications. It is also demonstrated that they have the ability to establish a prominent presence among other performance materials. These advanced polyesters have unique structures due to their amorphous and semi-crystalline nature, which results in exceptional performance behavior such as thermal, mechanical, optical, and barrier (water and thermal barrier) characteristics, making them suitable for use in textiles, packaging, and flexible electronics.

This review paper also discusses the role of structure–property relationships in determining the desirable performance characteristics of the resulting polymers. It has been demonstrated that the addition of a second diol or diacid to the primary backbone of the molecular chain considerably improves the performance of synthesized copolyesters, indicating that they are adaptable for various industrial applications. Furthermore, the importance of monomer stereochemistry in maximizing material qualities is emphasized, allowing for bespoke solutions to specific applications.

The exploration of various polymer synthesis approaches, such as solution, melt, and solid-state polymerization, provides significant insight into the synthesis of these innovative polymeric materials. The potential for using sustainable and biodegradable cyclic monomers, as well as green synthesis approaches, represents a promising direction for future research, in line with the increasing focus on environmental sustainability.

Even though cyclic monomer-based polyesters and CHDM have made significant strides, there are still issues that need to be addressed, such as the need for more thorough research on the synthesis and characterization of these materials, the development of more effective and environmentally friendly manufacturing techniques, and the search for novel cyclic monomers with improved properties.

The next generation of smart film applications will benefit greatly from advancements in cyclic monomer-based advanced polyesters and CHDM. Future research should concentrate on enhancing features such as transparency, flexibility, and thermal stability by engineering the polymer backbone structures to establish a balance between amorphous and semi-crystalline phases, as well as investigating environmentally friendly synthesis pathways and sustainable supply.

Overall, this comprehensive review article has provided a complete overview of the current state of CHDM and cyclic monomer-based polyesters for use in smart film applications. It is expected that the knowledge gained from this review will stimulate further study and research in this area, eventually leading to the development of novel materials that can meet the changing needs of diverse sectors, such as construction, electrical and electronics, medical and healthcare, packaging, and textile industries, among others. Academia and industry must work together to overcome difficulties and realize the full promise of these enhanced polyesters in smart film applications [114].

## Figures and Tables

**Table 1:** Summary of various preparation methods of 1,4-CHDM.

Sr. No.	Catalyst	Summary	Yield (%)	Ref.
1	physically mixed Pd-Cu-based	This research work involves the one-pot synthesis of CHDM from DMT using mixture of Pd-Cu as an active catalyst. The step of the conversion process is discussed in detail, and the impact of catalysts is studied in detail.	82	[48]
2	Ru/AL_03_ for 1st step CuO/Cr_203_ 2nd step	This work indicates the 2-step conversion process of DMT into CHDM in the presence of suitable solvent, methanol. Initially, DMT is converted into dimethyl cyclohexanedicarboxylate in excess solvent and then final product, and CHDM is achieved in the subsequent process. Various factors that affect the yield of the CHDM synthesis are also highlighted.	98	[49]
3	CuMnAl	Focuses on the use of palladium catalysts for the hydrogenation of DMT to CHDM, emphasizing the role of catalyst preparation and reaction conditions in achieving high yields.	95	[50]
4	Pd	This work highlights the synergic effect of palladium catalysts for the preparation of CHDM from DMT, a starting chemical. In order to achieve high yields, it also emphasizes the significance of catalyst preparation and reaction conditions.	95	[51]
5	Rh/Al_2_O_3_	This work focuses on the effects of reaction parameters on the yield of CHDM during the hydrogenation of DMT using rhodium on alumina (Rh/Al_2_O_3_) catalysts.	88–93	[52]
6	Cu_1.55_/Mg_2.45_Al_2_O_7_	This work focuses on the high-yield process (98%) for CHDM synthesis in the presence of Cu_1.55_/Mg_2.45_Al_2_O_7_ catalyst. The catalyst was highly efficient in the hydrogenation of BHCD to CHDM. The high selectivity and activity of the catalyst were attributed to its high dispersion and basicity.	98	[53]
7	Ru_4_Pt_2_Sn_8_/Al_2_O_3_	This study investigates the use of supported trimetallic RuPtSn/Al_2_O_3_ catalysts in the one-pot conversion of dimethyl terephthalate (DMT) to 1,4-cyclohexanedimethanol (CHDM). This study emphasizes how well the catalysts work to achieve high levels of efficiency and selectivity throughout the hydrogenation process. For CHDM, the reported response yield is almost 92%.	92	[54]
8	reduced graphene oxide (Pd/r-GO) and oxalate-gel-derived copper-zinc oxide (og-CuZn)	This work represents the novel 2-step process for the synthesis of trans-isomer-enriched 1,4-CHDM, an essential constituent of specialty polymers, from PET, using dual catalysts, Pd/r-GO and og-CuZn. The yield of the reaction was 95%. Furthermore, the isolated yield of 87% and a high trans/cis ratio on a 10 g scale was achieved, and the process efficiently transforms post-consumer PET plastics with yields ranging from 78% to 89%. This method increases selectivity in catalytic processes and provides a sustainable path for PET recycling.	95	[55]
9	Cu/Zn/Al	The present research proposed the synthesis of CHDM, 1,4-cyclohexanedicarboxylic acid, and 1,2-cyclohexanedicarboxylates using formaldehyde, crotonaldehyde, and acrylate/fumarate as starting materials. This work investigates a unique method for producing these compounds, which are crucial intermediates in the synthesis of polymers, using catalytic hydrogenation processes. This study emphasizes the catalytic system’s efficiency and potential for industrial use.	85	[46]
10	Re/AC (rhenium on activated carbon)	The purpose of this work is to investigate the selective hydrogenation of dimethyl 1,4-cyclohexanedicarboxylate to 1,4-cyclohexanedimethanol (CHDM) using Re/AC catalysts. It highlights how important the chemical environment and metal dispersion are to obtaining good catalytic activity. This study shows that improving these variables improves the hydrogenation process’s efficiency and selectivity.	97	[56]

**Table 2:** Unique characteristics and applications of various CHDM-based copolyesters.

Sr. #	CHDM Based Polyester	Unique Characteristics	Suitable Applications Area	Ref.
1	Poly(1,4-cyclohexandimethanol terephthalate (PCT)	High molecular weight and high melting and glass transition temperature	High temperature fiber, textile, electrical connectors	[71]
2	Isophthalic acid-modified (IPA) PCT (PCTA)	Tune-able processing properties, wide processing window, and amorphous nature suitable for molding applications	Injection molding applications, Extrusion molding applications, High-temperature applications such as electronic connector edge card connectors, grid arrays, memory modules, thermoformed trays for foods, transparent toys, and monofilament in paper machine belts	[71]
3	Glycol-modified amorphous PCT copolyesters (PETG/PCTG)	Transparent and glassy surface, ease of processing, good mechanical properties, and biocompatible	Mug container, crisper, heavy gauge sheet products, blister packages, and medical devices with good resistance to lipid solutions	[14,71]
4	1. Poly(butylene cyclohexanedimethylene succinate terephthalates) (PBCSTs) 2. Poly(butylene succinate-co-cyclohexanedimethanol succinate)	Moderate melting temperature, partially biodegradable, acceptable resistance against hydrolysis, tune-able, good thermal stability, etc.	Biodegradable packaging, textiles and fibers, agricultural mulch films, etc.	[58,72]
5	Poly(butylene 1,4-cyclohexanedicarboxylate) (PBCE)	Resistance against hydrolysis and environmental stress cracks, biocompatible with limited biodegradability, etc.	Tissue engineering, engineering plastic, and flexible packaging film	[73]
6	Cycloaliphatic diesters: dimethyl-1,4-cyclohexane: dimethyl-1,4-cyclohexane dicarboxylate (DMCD), dimethyl bicyclo heptane-1,4-dicarboxylate (DMCD-1), dimethyl bicyclooctane-1,4-dicarboxy-late (DMCD-2), dimethyl bicyclononane-1,5-dicarboxylate (DMCD-3), 1,4-dimethoxycarbonyl-1,4-dimethylcyclohexane (DMCD-M), and the aliphatic diols: ethylene glycol (EG) and 1,4-cyclohexane dimethanol (CHDM)-based poly[x(DMCD-2)y(DMCD) 30(EG)70(CHDM)] copolyester	Good optical, mechanical, chemical, and thermal stability; good glass transition temperature and flexibility; etc.	Outdoor applications, stable substrates for flexible displays and solar cells, and used as substrates for microfluidic devices	[49]
7	Poly (Ethylene Glycol 1,4-Cyclohexane Dimethylene Isosorbide Terephthalate) and Poly (1,4-Cyclohexane Dimethylene Isosorbide Terephthalate)	Moderate glass transition temperature along with acceptable melting temperature, good toughness and flexibility, good resistance against hydrolysis, chemicals and solvents, etc.	Tissue engineering, separators, filtration, and scaffolding	[74]
8	poly(1,4-cyclohexanedimethylene-co-isosorbide terephthalate)	Good thermal stability with high glass transition temperature, partially sustainable, resistance against hydrolysis and environmental stress cracks, etc.	Transparent rigid packaging, biodegradable and sustainable plastics, and medical and healthcare devices	[75]
9	poly (Ethylene-glycol-co-1, 4-cyclohexane dimethylene-co-isosorbide terephthalate)	Moderate to high glass transition temperature, biocompatible, good resistance to hydrolysis especially in humid environment, etc.	Artificial scaffold and artificial blood vessel	[76]
10	Poly(terephthalate-co-1,4-cyclohexanedimethanol/1,4-bis(hydroxymethyl)cyclohexane diester)	Moderate to high glass transition temperature, good thermal stability, high tensile strength and rigidity, good optical and chemical resistance properties, etc.	Durable household, kitchen, dishwasher container, and electronic device parts	[77]
11	Poly(cyclohexanedimethylene isosorbide terephthalate-co-naphthalate)	High glass transition, wide processing window, good water barrier, good thermal and dimensional stability, and good optical properties	Transparent substrate for flexible electronic devices and flexible packaging film	[78]
12	Poly(1,4-cyclohexanedimthylene terephthalate-co-1,4-cyclohxylenedimethylene 2,6-naphthalenedicarboxylate)	Good thermal degradation, good water barrier and optical behavior, and low coefficient of thermal expansion	Next-Generation Smart Film application and electronics packaging	[79]

**Table 3:** Summary of various polymer-synthesis techniques.

Sr. #	Synthesis Technique	Pros.	Cons	Ref.
1	Solution polymerization This is a process in which all the monomers are reacted together in the presence of suitable catalyst or/and stabilizers. These chemicals and resultant polymer are dissolved in suitable solvent. The solvent facilitates heat transfer along with the control of the viscosity of the reaction mixture. The final polymeric product is dissolved in the solvent, which is recovered by suitable filtration or separation process.	Ease of synthesis, uniform and good control over the reaction temperature, suitable for various polymer synthesis that have high melt viscosity, ease of separation of final product from solvent, etc.	Requires solvent-recovery system, environmental concerns due to solvent, low yield of polymer-synthesis process, risk of the presence of solvent in the final product, significant amount of energy is required (making it energy-intensive process), etc.	[92,107,108]
2	Melt polymerization This is the process in which two or more monomers react together in the presence of suitable initiator/catalyst and stabilizer. In this process, molten monomers react together under inert conditions in the absence of any solvent. The molten product is achieved at the end of the process.	Solvent-free synthesis process, high-molecular and pure product, energy efficient as no solvent extraction system is required, simple and straight forward process, high yield, etc.	High synthesis temperature is required, which sometime leads to thermal degradation of monomers/product; high viscosity of the products leads to difficulty in handling (especially at the extrusion from reactor stage); if reaction conditions are not optimized, it leads to incomplete polymerization.	[92,108,109,110]
3	Solid state polymerization (SSP) This is the process that is generally carried out after the polymer synthesis is at a suitable temperature between glass transition temperature and melting temperature. In this process, polymer undergoes heating, which promotes the polymerization and improves the cross-linking that leads to the increased molecular weight of the final product.	High-molecular-weight product, no need of any kind of toxic solvent, improved thermal, mechanical, and degradation stability characteristics, energy efficient and controlled process.	This is slow process generally completed in 24 h; risk of thermal degradation of polymeric pallets; it is not applicable to all polymers, and special equipment coupled with inert atmosphere is required; and there is challenge in uniform distribution of heat during the process, etc.	[105,111]

**Table 4:** Chemical composition and comparative properties of PET, PCT, PEN, and PCN homopolymers [24,26,36,39,40].

Properties	PET [24]	PCT [36]	PEN [26]	PCN [39,40]
Monomers	EG, TPA	CHDM, TPA	EG, NDA	CHDM, NDA
T_g_ (°C)	80	88	122	139
T_m_ (°C)	260	297	269	320
T_c_ (°C)	140	-	189	-
M_w_	44,800	53,200	50,600	49,195
M_n_	20,600	23,600	23,600	28,821
Polymer disparity index	2.17	2.25	2.14	1.71
Lattice structure	Triclinic	Triclinic	Triclinic	triclinic
Density (g/cm^3^)	1.337	1.197	1.198	1.313
Intrinsic Viscosity (dL/g)	0.70	0.79	0.84	0.70
Young’s modulus, MPa	3900	3660	5200	-
Tensile strength, MPa	45	52	60	-
Break elongation, %	150	250	65	-
UV absorbance (360 nm, %)	1	0.90	17	-
Oxygen permeability (cm^3^-mil/100 in.^2^-24 h-atm)	9.0	40	3.1	1.47
Hydrolysis resistance, h	50	-	200	-

**Table 5:** Thermal properties of PCTN# copolymers after SSP [92].

Samples	T_g_ (°C) ^a^	T_m_ (°C) ^b^	∆Hm (J g^−1^) ^c^	Crystallinity (%)	Reference
PCTN_0	83.79	285.76	48.53	47.6	[92]
PCTN_18	84.75	261.69	31.94	31.3	[92]
PCTN_26	85.12	246.81	31.43	30.81	[92]
PCTN_36	85.35	235.85	39.49	38.7	[92]
PCTN_47	88.62	238.21	35.54	34.8	[92]
PCTN_56	92.62	255.17	27.16	26.6	[92]
PCTN_65	107.54	278.61	28.33	27.8	[92]
PCTN_83	114.30	310.21	39.37	38.6	[92]

^a,b,c^ All the results of DSC from 2nd-run cycle (heating—quenching—heating).

**Table 6:** Thermal properties of acid-modified copolyesters (PCTAs) [36,82,126].

Copolyester Composition	T_g_ (°C)	T_m_ (°C)	IV (dL g^−1^)	Reference
PCT	88.0	295.3	0.85	[36]
PCTA-48	66	225	0.75	[125]
PCTN_30	97.91	244.88	0.79	[82]
PCTN_70	110.28	279.76	0.76	[82]
PCTS_17	-	286.0	1.04	[126]
PCTS_25	-	280.0	0.93	[126]
PCTSA_17	-	268	0.91	[126]
PCTSA_25	-	270	0.94	[126]
PETg_30_N-30	85	-	0.73	[126]
PETg_30_S-30	44	-	0.65	[126]

A: CHDA (95% trans), N: 2,6-NDA, S: succinic acid, SA: sebacic acid.

**Table 7:** Basic properties of conventional materials used as flexible substrate materials for displays [135,136,137].

Property	PET (Malinex) [135,136,137]	PEN (Teonex) [135,136,137]	Glass [136]	PI (Kapton) [135]	Steel [136]
Optical property (% transmission for 400–700 nm)	>85	0.85	>92	yellow	0.0
T_g_ (°C)	80	121	-	410	-
Water absorption (%)	0.4	0.4	0.0	1.8	0.0
Permeable to oxygen	yes	yes	no	yes	no
Young’s modulus (GPa)	5.3	6.1	80	2.5	200
Tensile strength, (MPa)	225	275	27–62	231	370
CLTE −55 to 85 °C (ppm/°C)	15	13	4	30–60	10
Maximum processing temperature (°C)	80	180	600	300	1000
Deform after device fabrication	yes	yes	no	yes	no
Roll to roll processing?	likely	likely	unlikely	likely	yes
Prebake required?	yes	yes	maybe	yes	no
Electrical conductivity	none	none	none	none	high
Upper working temperature	115–170	155	600	250–320	1400
Thermal conductivity (W/m °C)	0.1	0.1	1	0	16
Safe bending radius (cm)	-	4	40	4	4
Refractive index	1.66	1.75	1.52	1.50	2.76
Coefficient of hydrolytic expansion (ppm/%RH)	-	11	0	11	0
Thermal conductivity (W/m °C)	0.1	0.1	1	0.1–0.2	16
Density g/cm^3^	1.4	1.36	2.70	1.43	7.8

**Table 8:** Comparative analysis of the key performance characteristics of advanced polymeric substrates in comparison to conventional PI polymeric substrates [78,79,124,135,140].

Property	PI (Kapton) [135]	PCTN (Uniaxially Stretched) [79]	PCTN (Biaxially Stretched) [140]	PCITN (Randomly Oriented) [124]	PCITN (Uniaxially Stretched) [78]
Glass transition temperature (°C)	360	127	124.3	120.4	140
Melting temperature (°C)	-	279	276.8	279	275
Commercial availability	Yes	No	No	No	No
Transmission (300–800 nm), %	Yellow	87	94	86.7	86
CTE (−55 to 85 °C) (ppm °C^−1^)	30–60	6.0	13.6	-	5.8
Young’s modulus (GPa)	2.5	2.1	2.8	2.2	2.6
Birefringence (△n)	-	0.09	0.003	0.08	0.09
Water absorption (%) (Randomly Oriented)	1.8	0.37	0.16	0.21	0.14

## Abbreviations

CHDM	1,4-Cycloheanedimethanol	PET	Polyethylene Terephthalate
PEN	Poly(ethylene Naphthalene 2,6-dicarboxylate)	NDA	2,6-Naphthalenedicarbxylic Acid
PCT	Poly(1,4-cyclohexanedimethylene terephthalate)	TPA	Terephthalic Acid
IPA	Isophthalic Acid	PCTA	acid-modified PCT
DMT	Dimethylene Terephthalate	DMCD	Dimethyl Cyclohexanedicarboxylate
CHDA	1,4-Cyclohexanedicarboxyklic Acid	PCCD	Poly(1,4-cyclohexylene 1,4-cyclohexanedicarboxylate)
PBCC	Poly(butylene-co1,4-cyclohexanedimethylene carbonate)	ISB	Isosorbide
LCP	Liquid Crystalline Polymers	PCTN	Poly(1-4,cyclohexane dimethylene terephthalate-co-naphthalene dicarboxylate)
ROP	ring-opening polymerization	coPExCyT	Poly(ethylene-co-1,4-cyclohexanesimethylene terephthalate) copolyesters
SSP	solid state polycondensation	Tc	Cold Crystallization Temperature
Tm	melting temperature	PCN	Poly(cyclohexane dimethylene naphthalene dicarboxylate)
Mw	Weight Average Molecular Weight	Mn	Number Average Molecular Weight.
PEN-co-CN	Poly(ethylene 2,6-naphthalte-co-1,4-cyclohexanedimethanol 2,6-naphthalate)	PBN-co-CN	Poly(butylene 2,6-naphthalate-co-1,4-cyclohexanedimthylene 2,6-naphthalate)
PHN-co-CN	Poly(hexamethylene 2,6-naphthalate-co-1,4-cyclohexanedimthylene 2,6-naphthalate)	PTCT	Poly(trimethylene-co-1,4-cyclohexanedimethylene terephthalate)
PETG	Poly(ethylene glycol-co-1,4-cyclihexanedimethanil terephthalate)	P(CT-co-HT)	Poly(1,4-cyclohexylenedimethylene terephthalate-co-hexamethylene terephthalate)
PCTIN	Poly(1,4-cyclohexane dimethylene isosorbide terephthalate naphthalate)	N	2,6-NDA.
S	succinic acid	SA	sebacic acid
PECFs	Poly(ethylene 2,5-furandicarboxylate).	PCN	Poly(1,4-cyclohexanedimethylene 2,6-naphthalene)
TFTs	Thin Film Transistor.	OLEDs	Organic Light-Emitting Diodes
Tmax	Fabrication Process Temperature.	PC	Polycarbonate
PES	Polyethersulphone	PAR	Polyarylate
PI	Polyimide	PCE	Poly Cyclic Olefin
CTE	Coefficient of Thermal Expansion		
